# Threat driven modeling framework using petri nets for e-learning system

**DOI:** 10.1186/s40064-016-2101-0

**Published:** 2016-04-14

**Authors:** Aditya Khamparia, Babita Pandey

**Affiliations:** Department of Computer Science and Engineering, Lovely Professional University, Phagwara, Punjab India; Department of Computer Applications, Lovely Professional University, Phagwara, India

**Keywords:** Threat modeling, AOSPNs, Security metrics, Petri nets, Aspects, e-Learning

## Abstract

Vulnerabilities at various levels are main cause of security risks in e-learning system. This paper presents a modified threat driven modeling framework, to identify the threats after risk assessment which requires mitigation and how to mitigate those threats. To model those threat mitigations aspects oriented stochastic petri nets are used. This paper included security metrics based on vulnerabilities present in e-learning system. The Common Vulnerability Scoring System designed to provide a normalized method for rating vulnerabilities which will be used as basis in metric definitions and calculations. A case study has been also proposed which shows the need and feasibility of using aspect oriented stochastic petri net models for threat modeling which improves reliability, consistency and robustness of the e-learning system.

## Background

Due to enhancement in security problems for e-learning systems (Hecker 2008), it is essential that security concerns to be addressed in early stages of system development cycle (Jalal et al. 2008). Various e-learning systems are designed which are based on formal techniques and provides threat modeling only in requirement phase but not in design and analysis phase of existing system. Due to this there will be no guarantee that design vulnerabilities of system can be removed easily.

A petri net is one of mathematical modeling language or tool used for description of discrete distributed systems. It is a directed bipartite graph in which the nodes represent transitions, places and directed arcs (Murata 1989). It is a graphical based model used for stepwise processes which includes choice, iterations, and executions. Petri nets performed process analysis by using theory based on mathematical cases. Various types of petri nets are used to model behavior of system like colored petri nets (Houmb and Sallhammar 2012), timed petri nets and stochastic petri nets. Stochastic Petri Nets (SPNs) models distributed computing architectures and other software (Peterson 1977).

The proposed paper uses threat modeling for threat identification in system and categorizes those threats according to their categories (STRIDE) like spoofing identify, tampering data, repudiation, information disclosure, denial of service and elevation of privilege (Howard 2003). In proposed framework, new phases of threat modeling were added to fit with aspects and SPNs. Threat modeling offers various benefits such as (1) easier for team members to understand their application in better way; (2) easier to identify faults in system; (3) complex design faults can be identified easily which was not able to retrieve earlier in easy way.

The main system functions are modeled using SPNs whereas the threat mitigations are modeled using aspect oriented stochastic petri nets (AOSPN) which we have developed in this proposed research. Our modified threat driven framework measures the correctness, soundness and completeness of the SPN and AOSPN models (Dehlinger and Nalin 2006). Threat analysis (risk assessment), disintegration correction assessment, mitigation (attenuation) correction assessment and mitigation (attenuation) assessment are introduced phases that were added to threat modeling framework. In risk analysis phase risk of threat is measured by assigning the likelihood of occurrence and impact to system. Correctness assessment is measured using three main behavioral criteria of petri nets which are reachability, boundness and liveness. Mitigation (attenuation) assessment is calculated using a security metric that was adapted in this proposed work. Augmented metric is based on CVSS (Mell and Romanosky 2007) and proposed methods of Wang et al. (Wang et al. 2009). A modification in weight metric score was given to compute a quantitative score after applying the mitigations. On the basis of security metrics calculation (Payne 2006), we are able to observe how effective the mitigations were which enable e-learning researchers to compare mitigation effectiveness.

The rest of paper is organized as follows. “Literature review” section describes related work. “Aspect oriented SPN model (AOSPN)” section deals with aspect oriented SPNs. “Modified threat driven modeling framework” section describes the modified proposed threat framework model. “Proposed security metric” section shows the extended security metric and its calculations. “Case study” section deals with systematic case study, applying the threat driven framework and security metrics to specific question answer system and shows performance evaluation with respect to other frameworks. “Conclusion” section concludes the paper.

## Literature review

The proposed e-learning system mitigates the threats by performing threat modeling, aspect oriented development, usage of stochastic petri nets and security metric computation. The threat modeling is used to identify the threats which require mitigation and how to mitigate them. The process starts by disintegrating the applications, then determining and rank threats. Adapt methodology to respond to threats, choose best possible way to mitigate the threats and finally choose the appropriate technologies for the identified techniques.

Dehlinger and Nalin (2006) developed an aspect oriented model which provides UML based security and includes security policies as an aspect while designing a secure system. They have reviewed a security framework whose purpose is to provide the authors lessons derived from its design and use. They have verified the security of software using aspect oriented nets (Xu and Nygard 2006a, b). Their approach distinguished the software modeling and threat mitigations which are modeled by petri nets and aspect oriented nets simultaneously.

Sometimes the behavior of model not only depends on its structure but also on the timing. There is a requirement of stochastic petri nets (SPNs) which adds non deterministic time through adjustable randomness of the transitions (Haas 2002). These nets are modeled on basis of exponential random distributions and their performance analysis is based upon Markov theory (Balogh and Turcáni 2011). SPNs offers numerous advantages over original petri nets like ease of functional behavior analysis and testing with aid of graphical format, describe concurrency, synchronizations and show correlation among activities which describes the qualitative and quantitative properties of specified system like number of tokens firing from one place, how many tokens are expected to reach from one state to another at given time duration etc.

Over the last few years, developing methods to measure security loop holes is biggest challenge and concern among researchers. The NIST provided a paper as an overview of the security metrics area and looks at the possible possibilities of research that could be followed to advance the state of art (Jansen 2009). Some researchers distinguished between low level metrics and high level metrics for performing various estimations related to security. (Jensen 2008) created a tool SODAWeb which adapts and filter security techniques by using various applications supported by tools. (Heyman et al. 2008) have presented method of using security patterns to combine security metrics.

In our proposed security model, we have considered Common Vulnerability Scoring System (CVSS) (Mell and Romanosky 2007) which consists of three groups: Base, Temporal and Environmental. A numeric score has been produced by individual groups ranges from 0 to 10. A new approach was proposed by (Wang et al. 2009) to define software security metrics based on vulnerabilities included in software systems and their impacts on quality of software. We have utilized the approach in e-learning based systems. It uses the Common Vulnerabilities and Exposures (CVE) and CVSS in their metric definition and calculation. A complete comparative view of similarity and differences of proposed method with existing methods are given in Table 1.

**Table 1:** Similarity and differences of proposed method with existing models

Author	Proposed method	Similarity	Differences
Dehlinger and Nalin (2006)	Developed aspect oriented model to provide UML based security feature	They have used Aspect oriented net	The approach distinguished the software modeling and threat mitigations but without consideration of CVSS features
Omrani et al. (2011)	Proposed an adaptive e-learning system based on high level petri nets by considering learners learning style, score and knowledge level	They also evaluated the performance of e-learning system	They considered high level petri nets (HLPN) for performance evaluation without considering threats in system but in our security based model we have used stochastic petri nets (SPN) to improve robustness of system by using before and after mitigation strategy which improves learning performance
Balogh et al. (2012a, b)	Designed petri net based LMS system to regulate the communication according to student knowledge and ability and deliver learning material according to their needs	NA	They have focused on personalization using petri nets without consideration of security metrics which improves consistency and reliability of e-learning system
Hammami and Mathkour (2013)	Develop an e-learning system architecture which includes multi agent system and adaptive e-learning	NA	They have used object petri nets to build multi-agent architecture which adapts learner according to the learning preference and controls the communication and interaction among different agents. But in our proposed model we have used aspect oriented and stochastic petri nets with consideration of security, threat and risk assessment which has not been considered by Hammami and Mathkour (2013)

## Aspect oriented SPN model (AOSPN)

It incorporates the fundamental features of aspect oriented development. Aspects are the units that modularize the cross cutting concerns (cross cut the boundaries of traditional programming constructs). An aspect oriented program consists of a number of base modules and aspects that can be merged into an executable whole. AOSPN includes the basic concepts like join points, advices, pointcuts and introduction (Schauerhuber et al. 2006). An advice is contained by an aspect and is a piece of code that is inserted at one or more specific points of core concern. A join point is point in the execution where an advice is inserted. Join points may be transitions, predicates, and arcs in the SPN. A point cut is a language construct that designates a join point. Point cut defines whether a given join point matches according to defined criteria. An introduction net introduces new members to base modules. It allows aspects to modify the static structure of program.

In AOSPNs there are three types of pointcuts as described by: transition, predicate and arc. A stochastic petri net-based aspect A is a structure 〈P, D, I〉 where P is set of pointcuts, D is a set of advice nets and I is a set of introduction nets. Processing timed transition pointcuts remove all the transitions selected by each transition pointcuts and replace it with the corresponding introduction nets according to the advice specifications.

**Fig. 1:**
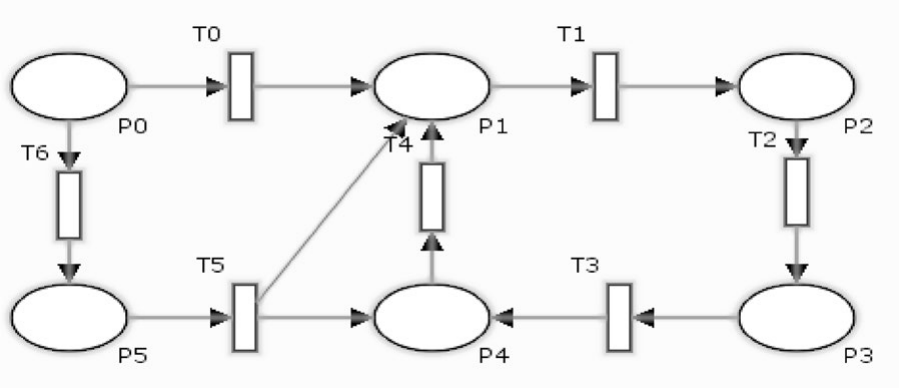
Stochastic petri net N1

**Fig. 2:**
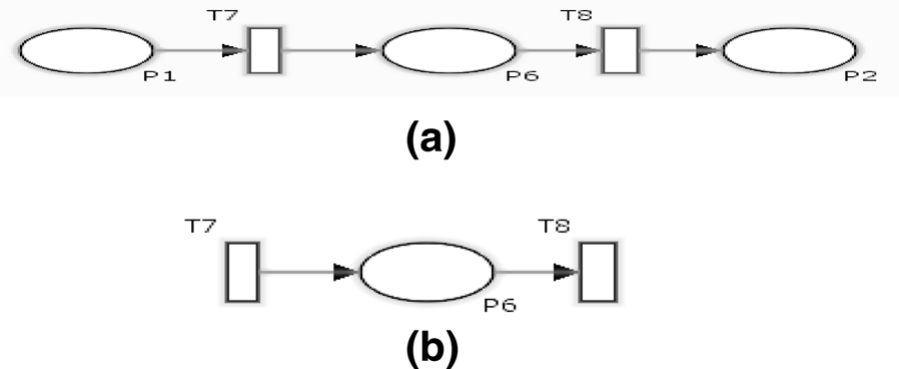
An aspect model with advice and introduction net. **a** Advice tcut. **b** Introduction net

Suppose there is a threat in the timed transition T1 in the stochastic petri net N1 in Fig. 1. We define the aspect as shown in Fig. 2 where the pointcut specifies the place of the threat, advice net described how the mitigation will be weaved and introduction net illustrates the mitigation. For clarity, the weaving mechanism assumes that a base net does not share names with SPNs in aspects. Aspects weaving with the base net results in a new stochastic petri net. It can further be weaved with other aspects that involve the original base net. The order in which aspects are applied to a base net is not significant.

AOSPN model alone cannot tackle the increasing challenge of lack of data, how a system may react to certain security attacks although the chances of future security attacks are still unknown. There is little information known about the motivation and behaviour of attackers at this stage. To identify the attack trends report and vulnerabilities bulletin information in terms of CVSS is known. The benefit of CVSS is that it addresses the vulnerabilities directly and in collaboration with the vendors of the affected products. That is, CVSS tries to be specific and do not attempt to categorize attacks on a general basis nor does it provide a general model for estimating risk level. CVSS purely provides information about vulnerabilities on an operational level and leaves it to the vendors to add the information specific for their products and to the customers to interpret the information in the perspective of a particular Target of Evaluation. It is always better to use environmental metrics along with base and temporal metrics which we incorporated in our approach as given in CVSS to evaluate integrity, availability and confidentiality rather than productivity, reputation and privacy (Houmb and Franqueira 2009).

## Modified threat driven modeling framework

**Fig. 3:**
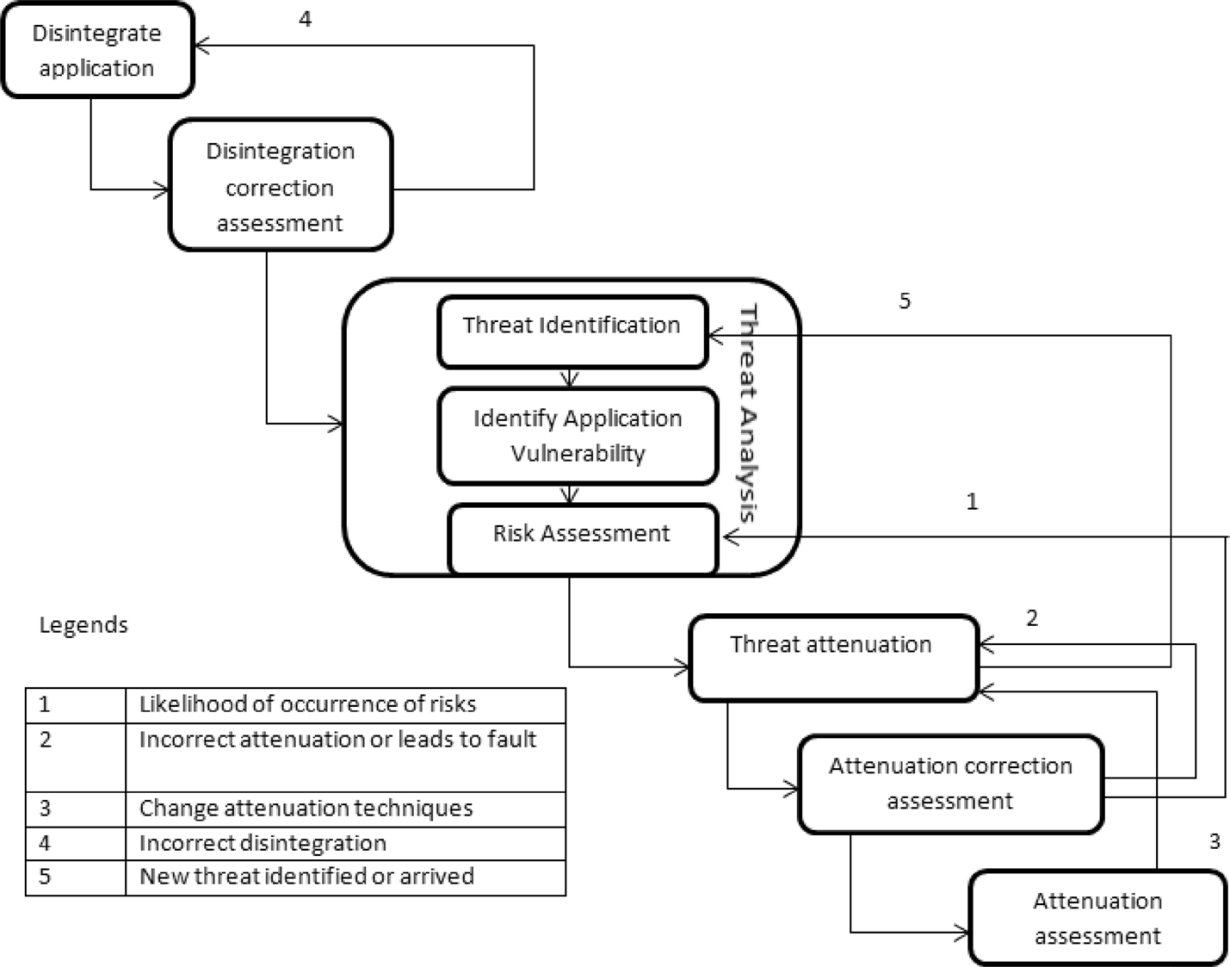
Threat driven modeling framework

The threat driven framework has been illustrated in Fig. 3 which is used to provide security in software based e-learning systems. This proposed framework comprises of six steps which are Disintegrate application (decomposition), Disintegration correction assessment, Threat analysis (Threat identification, Identify Application vulnerability, and Risk assessment), Threat mitigation, Mitigation correction assessment and Mitigation assessment.

Our framework has proposed some modifications over the threat driven framework proposed by (Shrief et al. 2010) and traditional framework. Out of these steps Disintegrate application, Threat identification and Threat mitigation are taken from traditional framework and framework proposed by (Shrief et al. 2010) as shown in (Howard 2003), while remaining steps were customized according to their usage with SPNs (Murata 1989; Peterson 1977; Haas 2002; Wang et al. 2009).

1. Disintegrate application: In this phase based on systems requirement the main module will be modeled using SPNs. The existing models using UML can be easily transferred to SPNs. Further SPNs utilized for system functions as deliverables.
2. Disintegration correction assessment: In this phase the behavioral properties for SPNs will be tested using basic properties of nets like Reachability, boundness, liveness and safeness. Due to changes in behavioral properties if the SPN leads to deadlock or starvation then changes can be made by reverting back to previous phase.
3. Threat analysis: This phase is carried out in three steps: Threat identification, Identification of application vulnerability and risk assessment. After disintegration phase threat has been identified and modeled through SPNs. Threat has been categorized using STRIDE in which identified threats marked on the SPNs as pointcuts. In next step security vulnerabilities for individual applications in e-learning were identified. Some of vulnerabilities are (authentication, authorization, input and data validation, configuration management, session management, auditing and logging etc.) for applications like virtual learning environment, student administration, mobile learning, virtual learning, certification etc. (Hayaati and Fan 2010). In last phase, the effect of threat can be identified on e-learning system using risk assessment. Threat matrix has been generated along with threats corresponding to vulnerabilities and prioritizes them on the basis of their likelihood of occurrence.
4. Threat mitigation: In this phase, the techniques to attenuate the threats are chosen. The deliverable of this phase is set of aspects describing the mitigations (introduction net) and how they will be inserted to original system with help of (advice nets) in specified pointcuts. If the new or unidentified threats occur after applying mitigation then they can be identified back in the previous phase i.e. threat analysis and then attenuated.
5. Mitigation correction assessment: In this phase if applying the mitigation leads to fault in the behavioral properties or due to incorrect mitigation then changes can be made by going back to previous phase and redesign the mitigations. After applying mitigation, if there is chance of likelihood of occurrence of risks then it can be redirected back to risk assessments phase in threat analysis to minimize threat affect in nets.
6. Mitigation assessment: This is a recurrent phase which will be repeated before and after mitigation in which various security metrics will be applied to determine the potency of selected threat mitigations. The system threat level will be indicated by numeric values. If there is no changes in results obtained by numeric values in decreasing order then that appropriate mitigation were not chosen. So better mitigation techniques will be selected further by returning back to previous phases.

## Proposed security metric

The proposed security metric is based on the CVSS (Common Vulnerability Scoring System) which is customization and modification of work done by Wang et al. The security steps were customized so that they can be used with SPN models and they relied on the weakness of the e-learning system software. The proposed security metric process has been carried out in eight steps as follows:

1. Identification of weaknesses and vulnerabilities in applications.
2. Calculate severity for individual vulnerabilities.
3. Calculate the probability of vulnerability occurrence.
4. Calculate the probability of threat occurrence and risk assessment.
5. Calculate the percentage of each weakness.
6. Calculate the security metric.
7. Again calculate threats severity after mitigation.
8. Recalculate the security metric.

Various equations have been used for depiction of security metrics. The security metric (SM(s)) is calculated by product of severity of weakness (W_n_) and risk of corresponding weakness (P_n_) as shown in Eq. 1. Here n = 1, 2, 3…m.

1 $$SM\left( s \right) = \mathop \sum \limits_{n = 1}^{m} \left( {P_{n } \times W_{n} } \right)$$

Now, W_n_ is defined as average base score of its k vulnerabilities, as shown in Eq. 2.

2 $$W_{n} = \mathop \sum \limits_{i = 1}^{k} \frac{{V_{i} }}{K}$$

The percentage each representative weakness occurs in the overall weakness occurrences is used to calculate P_n_ as shown in Eq. 3.

3 $$P_{n} = \frac{{R_{n} }}{{\mathop \sum \nolimits_{i = 1}^{m} R_{i} }}$$

where R_n_ is the frequency of occurrences for each representative weakness in the SPN as shown in Eq. 4, where K is the number of weaknesses and A is the sum of affected nodes in SPNs.

4 $$R_{n} = \frac{K}{{\mathop \sum \nolimits_{i = 1}^{m} A}}$$

To make the value of SM(s) value to range from 0 to 10 is required to hold for P_n_.

5 $$\mathop \sum \limits_{n = 1}^{n} P_{n} = 1$$

The severity of each weakness in e-learning systems after mitigation is recalculated as shown in Eq. 6. Here E denotes Exploitability, RL denotes Remediation Level and RC for Report Confidence which are important temporal metrics of CVSS. CR denotes Confidentiality Requirement, IR denotes Integrity Requirement and AR denote Availability Requirement which are environmental metrics of CVSS.

6 $$W_{{n_{new} }} = \mathop \sum \limits_{i = 1}^{k} \frac{{V_{i} \times E \times RL \times RC}}{K \times CR \times IR \times AR }$$

For each mitigation weakness, if there exists certain vulnerabilities that still occurred are identified by recalculating Eq. 4. If the number of affected nodes become same compared to the results obtained after applying mitigations then security metric has to be recalculated with help of Eq. 1. The proposed system intended to identify threats and their analysis in design and analysis phase, therefore the number of nodes affected in the SPN will be compromised due to threat occurrence is used. To re-compute the threat’s severity after applying the mitigation CVSS based Eq. 6 is added for solving computations.

## Case study

The proposed framework modules have been applied to case study on modeling of Question–Answer system for Udutu based e-learning system. (Shrief et al. 2010) developed their threat framework and applied their framework on AI specific question answering system which is different from our question answering system.

### Decompose application

The Question–Answer application in Udutu based system allows users to ask questions on Java Programming and system processes those questions (objective or subjective) and produces an answer. After user get authorized and authenticated by the system, he/she could enter question on Java modules. The system responsibility is to check whether there exists direct answer to that question or not. If direct answer exists, then it can be retrieved from knowledge base and displayed. Otherwise, system process the same question by searching the possible collective keywords to the nearest possible answers stored in knowledge base. From the available data, all possible answers have been created and from these answers select the best answer specified by user and finally display the appropriate answer.

**Fig. 4:**
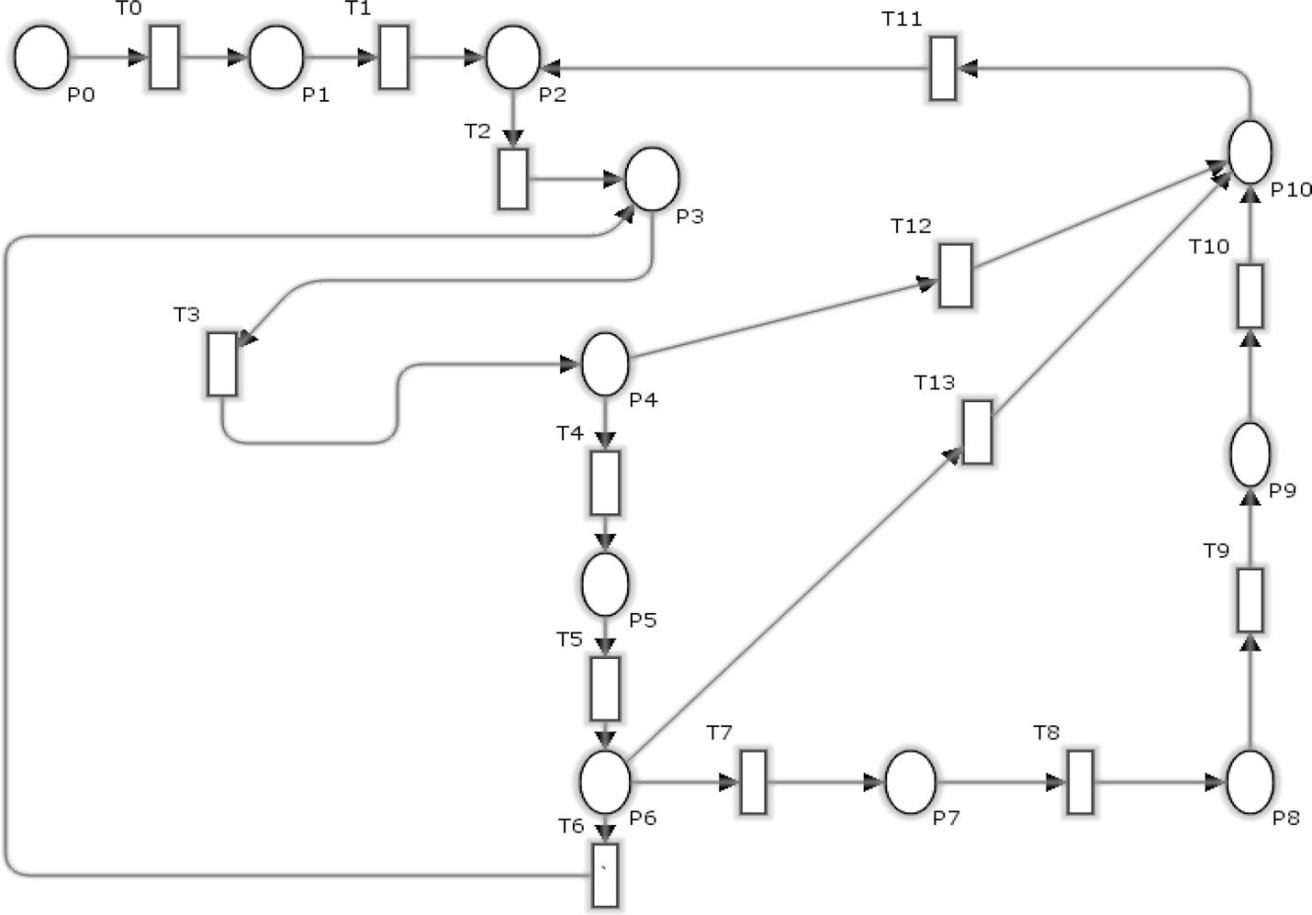
Petri net model for question answering system

The SPNs are best designed and modeled by Petri nets model which contain random events and perform processing of input data. The Petri net modeling is shown in Fig. 4 in which initial marking starts by one token in P0 that carries out different values throughout the transition firings from one place to another.

For better understanding of above depicted model, meanings of places and transitions are shown in Table 2 respectively.

**Table 2:** Place and transitions for question answer system

Place number	Place description	Transition number	Transition description
P0	User login	T0	Authenticate
P1	Authenticated/authorized	T1	Go to main page
P2	System ready state	T2	Enter an objective question
P3	Objective question asked	T3	Search if a direct objective answer exists
P4	Direct answer yes/no	T4	Enter a subjective question
P5	Subjective question asked	T5	Search if a direct subjective answer exists
P6	Direct answer yes/no	T6	Searching the data in objective question knowledge base
P7	Data found	T7	Create the answers
P8	Answer formed	T8	Select from the answers
P9	Answer selected	T9	Display an answer
P10	Response displayed	T10	Getting response from answers
		T11	Exit
		T12	Decrypt answer formation decision and retrieve direct response for objective
		T13	Decrypt answer formation decision and retrieve direct response for subjective

### Decomposition correction assessment

**Fig. 5:**
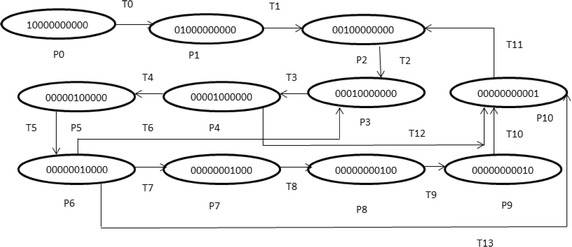
Reachability graph for SPNs of question answer system

To check correctness assessment for e-learning based system three main behavioral properties are required: reachability, boundness and liveness. Reachability determines whether a state can be reachable from one to other (Haas 2002). SPNs are k bounded if they doesn’t contain more than k tokens in all reachable markings, including initial marking. Liveness determines that any state which is reachable can be fired without coming into deadlock situation. The reachability graph shows in Fig. 5 shows the different markings and various states of SPNs that can be reached. This case study on e-learning based system is 1-bounded, live and also known as safe SPNs. The nodes in Fig. 5 show different markings while arcs are labeled with transition names to show that marking is reached by firing of certain transitions.

### Threat analysis

This module is divided into three phases as threat identification, application vulnerability and risk assessment.

### Identify threats

Various kinds of threats are used to be mitigated in present case study on e-learning system. First, when the user log into the system due to possibility of threats like network eavesdropping, password guessing, cookie reply there is chance to lead authentication vulnerability. Second, when system starts searching to check whether direct answer exists or not, an attacker can tamper the data and change the response formation mode. Third, an elevation of privilege can occur if an unauthorized user tries to decrypt the answer formation decision and search for data or display direct response. Finally, while creating possible answers from data gathered, an attacker can tamper the data and influence answer creation.

After identification of threats the security vulnerabilities for different applications in e-learning were identified then finally the threat is being analyzed by risk assessment matrix (Hayaati and Fan 2010). Risk of each threat is measured by assigning the likelihood of occurrence and impact to system. The risk evaluation done by using the risk evaluation grid proposed by (Barbeau 2005) Risks derived from threat analysis were classified in three main groups minor, major and critical which are decided by expert. The threat analysis result has been converted to the e-learning threats risk matrix as shown in Table 3.

**Table 3:**
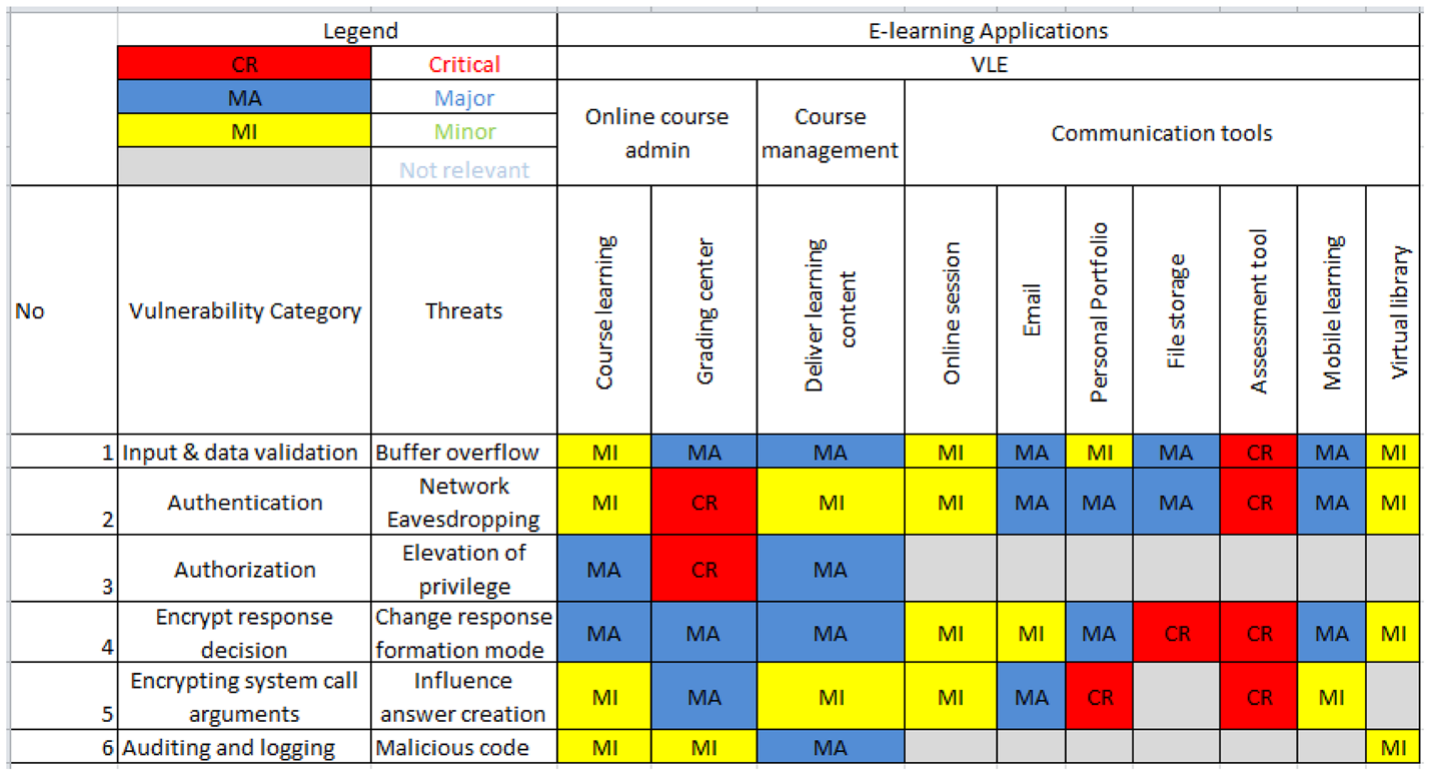
Threat matrix

*CR* critical, *MA* major, *MI* minor, – not relevant

### Mitigate threats

**Fig. 6:**
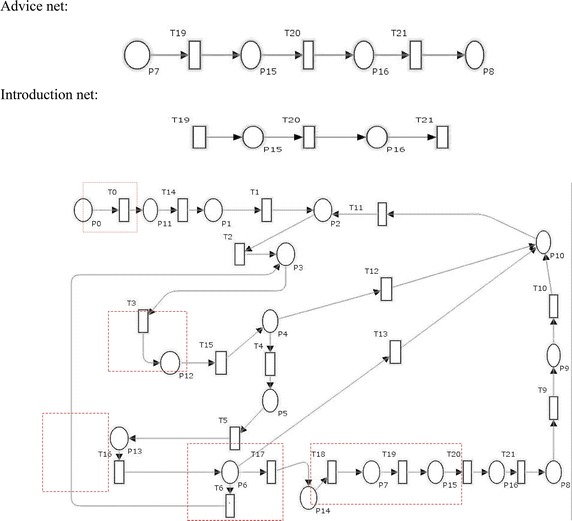
Petri net model after threat mitigation

Various types of threats have been identified and determined from categories of STRIDE. Here, as a sample the aspect for threat mitigation of tampering with data threat is provided. Some other threats can also be mitigated as shown as encircled in Fig. 6. The tampering with data threat is mitigated by encryption to prevent a code injection attack to influence the answer creation (Wang et al. 2009). As shown in aspect of threat in Fig. 6, T19 is the transition which represents the encrypting system call arguments (Oyama 2006); P15 represents the state of system where all calls are encrypted so that no attack can happen and tamper the data; P14 is process where arguments are decrypted after processing and then encrypted again; P16 is the system state where answer formation gets completed; and T21 is the process of decrypting the system call arguments. Other threats like elevation of privilege threat will be mitigated by authorization; similarly networking eavesdropping will be mitigated by authentication. For given aspect of data tampering threat the pointcut is T8; the advice net and introduction net will be

### Security metric calculation (mitigation correction assessment)

#### Identify weakness and vulnerabilities

Some of the important weaknesses in e-learning system were identified like network eavesdropping; change response formation mode; influence answer creation and elevation of privilege.

#### Calculate severity for each vulnerability

The CVSS for each vulnerability should be calculated by assigning values to each of the six base metrics and creating the base vector as follows:

1. Network eavesdropping: The base vector will be AV:[A]/AC:[H]/Au:[S]/C:[N]/I:[N]/A:[P] = 1.5
2. Change response formation mode: The base vector will be AV: [A]/AC:[H]/Au:[S]/C:[N]/I:[C]/A:[N] = 4.3.
3. Influence answer creation: The base vector will be AV:[A]/AC:[H]/Au:[S]/C:[C]/I:[C]/A:[P] = 6.2
4. Elevation of privilege: The base vector will be AV:[A]/AC:[H]/Au:[S]/C:[C]/I:[N]/A:[P] = 5.

#### Calculate the probability of vulnerability occurrence

The probability of vulnerability occurrence can be calculated by identifying the weakness and vulnerabilities occurrence in the software. These calculations are computed or obtained from Eq. 4 (R_n_). (1) Network eavesdropping: R_1_ = 1/20, (2) Change response formation mode: R_2_ = 1/20, (3) Influence answer creation: R_3_ = 1/20 + 1/20 = 1/10, (4) Elevation of privilege: R_4_ = 1/20 + 1/20 + 1/20 = 3/20.

#### Calculate the percentage of each weakness

The percentage of each weakness in the software is calculated from Eq. 2 (W_n_) and Eq. 3 (P_n_). (1) Network eavesdropping: P_1_ = R_1_/(R_1_ + R_2_ + R_3_ + R_4_) = 0.15, (2) Change response formation mode: P_2_ = R_2_/(R_1_ + R_2_ + R_3_ + R_4_) = 0.15, (3) Influence answer creation: P_3_ = R_3_/(R_1_ + R_2_ + R_3_ + R_4_) = 0.28, (4) Elevation of privilege: P_4_ = R_4_/(R_1_ + R_2_ + R_3_ + R_4_) = 0.42.

#### Calculate security metric

The outputs of Eqs. 2 and 3 are require to substituted in Eq. 1 to obtain the security metric value. The security metric score is calculated based on Eq. 1:

$$SM\left( s \right) = W_{1} \times P_{1} + W_{2} \times P_{2} + W_{3} \times P_{3} + W_{4} \times P_{4} = \left( {1.5 \times 0.15 + 4.3 \times 0.15 + 6.2 \times 0.28 + 5 \times 0.42} \right) = 4.7.$$

#### Recalculation of severity of threats after mitigation

For obtaining a comparative analysis between the state before and after mitigation the security metric SM(s) should be recomputed again. The resulting value obtained after computation should be less than the one computed before mitigations.

The CVSS temporal score should be calculated for each mitigated threat by assigning values to each of temporal metrics and created the temporal vector. The temporal score for the mitigations of four identified threats are:

1. Authentication: The temporal vector will be E:[F]/RL:[W]/RC:[C] = 1.35
2. Encrypt response decision: The temporal vector will be E:[POC]/RL:[W]/RC:[UR] = 3.5
3. Encrypting system call arguments: The temporal vector will be E:[H]/RL:[W]/RC:[C] = 5.9
4. Authorization: The temporal vector will be E:[F]/RL:[W]/RC:[C] = 4.5.

We have considered only confidentiality requirement (CR), integrity requirement (IR) and availability requirement (AR) (Heyman et al. 2008) metrics for calculation of $W_{{n_{new} }}$.

The environment metrics for identified threats are:

1. Authentication: The required environmental vector will be CR:[M]/IR:[H]/AR:[H].
  Where M is 1.0, H is 1.51.
2. Encrypt response decision: The required environmental vector will be CR:[H]/IR:[L]/AR:[M]
  Here M is 1.0, H is 1.51 and L is 0.5.
3. Encrypting system call arguments: The required environmental vector will be CR:[H]/IR:[H]/AR:[M].
4. Authorization: The required vector will be CR:[M]/IR:[H]/AR:[H].

From Eq. 6 the new obtained value for $W_{{n_{new} }}$ need to be calculated which gives new value for severity of weakness after applying mitigations as:

$$W_{{n_{new} }} = \frac{1.35}{{\left( {1 \times 1.51} \right)}} + \frac{3.5}{{\left( {1 \times 1.51 \times 0.5} \right)}} + \frac{5.9}{{\left( {1.51 \times 1.51 \times 1} \right)}} + \frac{4.5}{{\left( {1 \times 1.51 \times 1.51} \right)}}$$

Recalculate the security metric:

The security metric score SM(s) could be computed based on Eq. 1 after substituting $W_{{n_{new} }}$.

$$\begin{aligned} SM\left( s \right) & = P1 \times \frac{1.35}{{\left( {1 \times 1.51} \right)}} + P2 \times \frac{3.5}{{\left( {1 \times 1.51 \times 0.5} \right)}} + P3 \times \frac{5.9}{{\left( {0.5 \times 1.51 \times 1} \right)}} + P4 \times \frac{4.5}{{\left( {1 \times 1.51 \times 1.51} \right)}} \\ & = 0.15 \times 0.59 + 0.15 \times 4.63 + 0.28 \times 2.58 + 0.42 \times 1.97 = 2.33. \\ \end{aligned}$$

After evaluation of complete case study it was observed that before applying mitigations the threats determined in system and metric value was 4.7, whereas after applying the mitigations the threat mitigations the security metric was recomputed to check the effectiveness of the applied mitigations and scored metric value obtained was 2.33. It indicates that the mitigations were very effective in places where applied in system on basis of their occurrences. These security metric values indicate the effectiveness of applied mitigations and provide comparative analysis between different mitigations.

### Performance evaluation

We have compared our framework with two existing threat frameworks 1) traditional framework (Howard 2003) and framework proposed by (Shrief et al. 2010). The traditional framework only considered base metrics whereas the (Shrief et al. 2010) considered base and temporal metrics for the measurement of severity of threat. Our proposed framework is based on base, temporal and environment metrics therefore it gives better results as compared to the two existing frameworks. The comparative view of threat driven frameworks are shown in Table 4.

**Table 4:** Comparative view of various threat driven frameworks

Author	Modeling	Framework	Equation
Howard (2003)	NA	Framework consists of 3 modules: 1. Decompose application 2. Identify threats 3. Mitigate threats	After mitigation the severity is calculated only on basis of base metrics i.e. $W_{{n_{new} }} = \frac{{V_{i} }}{K}$
Shrief et al. (2010)	Stochastic petri net	Framework consists of 6 modules: 1. Decompose application 2. Decomposition correction assessment 3. Identify threats 4. Mitigate threats 5. Mitigation correction assessment 6. Mitigation assessment	After mitigation the severity is calculated only in terms of base and temporal metrics i.e. $W_{{n_{new} }} = \frac{{V_{i} \times E\times RL\times RC}}{K}$
Our proposed approach	Aspect oriented stochastic petri nets	Framework consists of 6 modules and threat identification is divided into sub modules. 1. Disintegrate application 2. Disintegration correction assessment 3.1. Threat identification 3.2. Identify application vulnerability 3.3. Risk assessment matrix 4. Mitigate (Attenuate) threats 5. Mitigation (Attenuation) correction assessment 6. Mitigation (Attenuation) assessment	After mitigation the severity is calculated only in terms of base, temporal and environmental metrics i.e. $W_{{n_{new} }} = \frac{{V_{i} \times E\times RL\times RC}}{K\times CR \times IR\times AR}$

## Conclusion

This paper has shown an effective security threat driven modeling framework, modified security metric with usage of CVSS and AOSPN models. In threat modeling framework correction assessment has been involved, mitigation correctness to measure the behavioral properties of SPNs and AOSPNs, and mitigation assessment to measure the mitigations effectiveness. These SPNs model weaved a point cut, advice nets and introduction nets into existing petri net system. Finally, security metric calculations were computed for SPNs with usage of CVSS and a new modified equation introduced by using base, temporal and environmental metrics to calculate the metric after mitigations to perform comparison among them.
